# Fibroblast growth factor signalling induces loss of progesterone receptor in breast cancer cells

**DOI:** 10.18632/oncotarget.13322

**Published:** 2016-11-12

**Authors:** Dominika Piasecka, Kamila Kitowska, Dominika Czaplinska, Kamil Mieczkowski, Magdalena Mieszkowska, Lukasz Turczyk, Andrzej C. Skladanowski, Anna J. Zaczek, Wojciech Biernat, Radzislaw Kordek, Hanna M. Romanska, Rafal Sadej

**Affiliations:** ^1^ Department of Molecular Enzymology, Intercollegiate Faculty of Biotechnology, University of Gdansk and Medical University of Gdansk, Poland; ^2^ Department of Cell Biology, Intercollegiate Faculty of Biotechnology, University of Gdansk and Medical University of Gdansk, Poland; ^3^ Department of Pathomorphology, Medical University of Gdansk, Poland; ^4^ Department of Pathology, Medical University of Lodz, Poland

**Keywords:** progesterone receptor, FGFR2, breast cancer

## Abstract

We have recently demonstrated that, fibroblast growth factor 2 (FGFR2), signalling via ribosomal S6 kinase 2 (RSK2), promotes progression of breast cancer (BCa). Loss of progesterone receptor (PR), whose activity in BCa cells can be stimulated by growth factor receptors (GFRs), is associated with poor patient outcome. Here we showed that FGF7/FGFR2 triggered phosphorylation of PR at Ser294, PR ubiquitination and subsequent receptor`s degradation via the 26S proteasome pathway in BCa cells. We further demonstrated that RSK2 mediated FGF7/FGFR2-induced PR downregulation. In addition, a strong synergistic effect of FGF7 and progesterone (Pg), reflected in the enhanced anchorage-independent growth and cell migration, was observed. Analysis of clinical material demonstrated that expression of PR inversely correlated with activated RSK (RSK-P) (*p* = 0.016). Patients with RSK-P(+)/PR(–) tumours had 3.629-fold higher risk of recurrence (*p* = 0.002), when compared with the rest of the cohort. Moreover, RSK-P(+)/PR(–) phenotype was shown as an independent prognostic factor (*p* = 0.006). These results indicate that the FGF7/FGFR2-RSK2 axis promotes PR turnover and activity, which may sensitize BCa cells to stromal stimuli and contribute to the progression toward steroid hormone negative BCa.

## INTRODUCTION

Progesterone receptor (PR), a ligand-activated transcription factor, belongs to the steroid hormone receptor family. Although it is encoded by a single gene (*PGR*), differential transcription, followed by translation from two alternative initiation codons, results in expression of two isoforms of the PR protein: PR A (90 kDa) and PR B (116 kDa) [1]. As demonstrated in rodents, a ratio of PR A/PR B expression is a key biological determinant of tissue responsiveness to ligand stimuli and it is a critical regulator of lobuloalveolar development of the mammary gland [2–4]. An involvement of PR in the initiation and progression of breast carcinoma (BCa) is indubitable [2], but the molecular mechanism of its action is complex and still remains poorly understood. An increased incidence of BCa in women taking both estrogen and progestins (synthetic ligands of progesterone (Pg)) for hormone replacement therapy, compared to estrogen alone [5], gives support to the impact of PR-mediated signalling on BCa pathophysiology. ER(+)/PR(+) tumours represent approximately 50–70% of all BCa cases, and PR loss is associated with resistance to hormonal therapy and increased tumour invasiveness [6], which is corroborated by numerous reports of substantially poorer outcome of patients with ER(+)/PR(−) tumours following tamoxifen-based treatment [7–10].

There are two types of PR signalling pathways: a classical (genomic), involving PR action in regulation of target gene expression, and an alternative (non-genomic), in which PR does not directly participate in gene transcription. In the classical pathway, upon ligand binding, cytoplasmic PR translocates to the nucleus and triggers expression of genes with the PRE (progesterone response element) sequence [11]. PR takes part in a large number of alternative, non-genomic signalling cascades in which PR activates MAPK and PI3K/AKT pathways rapidly initiating various cellular events (e.g. cell migration, adhesion, proliferation) or expression of related genes (*MSX2, RGS2, PDK4*) [12–15]. PR is highly post-translationally modified including phosphorylation, sumoylation, acetylation or ubiquitination [16, 17]. These modifications are frequently ligand-dependent but they might also occur independently of ligand-binding, for example, in response to kinases activity. PR modifications significantly alter receptor's stability, localization, transcriptional activity and promoter selectivity [18].

The mechanisms underlying acquisition of hormone independence by breast cancer cells still remain elusive. It is well recognised that reciprocal interactions between tumour cells and stromal microenvironment play a key role in cancer development. In particular, cancer-associated fibroblasts (CAFs) are considered to be crucial for tumour progression and metastasis [19]. By secretion of soluble factors such as transforming growth factor β (TGF-β), insulin-like growth factor-1 (IGF-1), stroma-derived factor 1 (SDF-1), or fibroblast growth factors (FGFs), CAFs promote tumour angiogenesis and increase invasiveness of cancer cells [20–22]. As demonstrated by Giulianelli et al., CAFs activate PR through paracrine action of FGF2, which induces hormone independent mammary tumour growth [23]. PR has so far been shown to be activated by growth factors (GFs) such as IGF-1 [24], epidermal growth factor (EGF) [16] and FGF2 [25]. Reciprocal interactions between PR- and growth factor receptors (GFR)-mediated signalling result in progesterone-independent activation of PR as well as PR-regulated GFR expression and function [25, 26]. Several studies demonstrated that activation of PR by phosphorylation at Ser294 is followed by nuclear localization and further receptor's degradation [18, 27–29]. This sequence is thought to be initiated by growth factors-triggered signalling, promoting generation of a pool of hypersensitive PR forms responsive to very low concentrations of the ligand [24].

Herein we showed for the first time that in MCF7, T47D and BT474 breast cancer cell lines, phosphorylation of PR at Ser294 and subsequent downregulation of PR protein level was induced by FGF7/FGFR2 (fibroblast growth receptor 2)-triggered signalling. Inhibition of proteasome 26S prevented FGF7-dependent PR downregulation suggesting that FGF7 signalling had led to PR proteolysis through the ubiquitin-related pathway. Furthermore, we showed that ribosomal S6 kinase 2 (RSK2) mediated FGF7/FGFR2-triggered PR downregulation in MCF7 cells. Analysis of clinical material demonstrated that expression of PR inversely correlated with activated RSK (RSK-P), thus confirming *in vitro* findings. Moreover, patients with RSK-P(+)/PR(–) tumours had worse disease-free survival (DFS) when compared to the rest of the cohort. In addition, FGF7 has been found to potentiate PR-dependent growth and migration of MCF7 cells. These results, together with our recently reported findings demonstrating that lack of combined immunoreactivity for FGFR2 and activated RSK (RSK-P) was predictive of a better patients’ DFS [30] suggest that FGF7/FGFR2 induces degradation and activity of PR which may contribute to microenvironment-driven shift of breast cancer cells towards hormone independence.

## RESULTS

### FGF7/FGFR2 action downregulates PR

**Figure 1:**
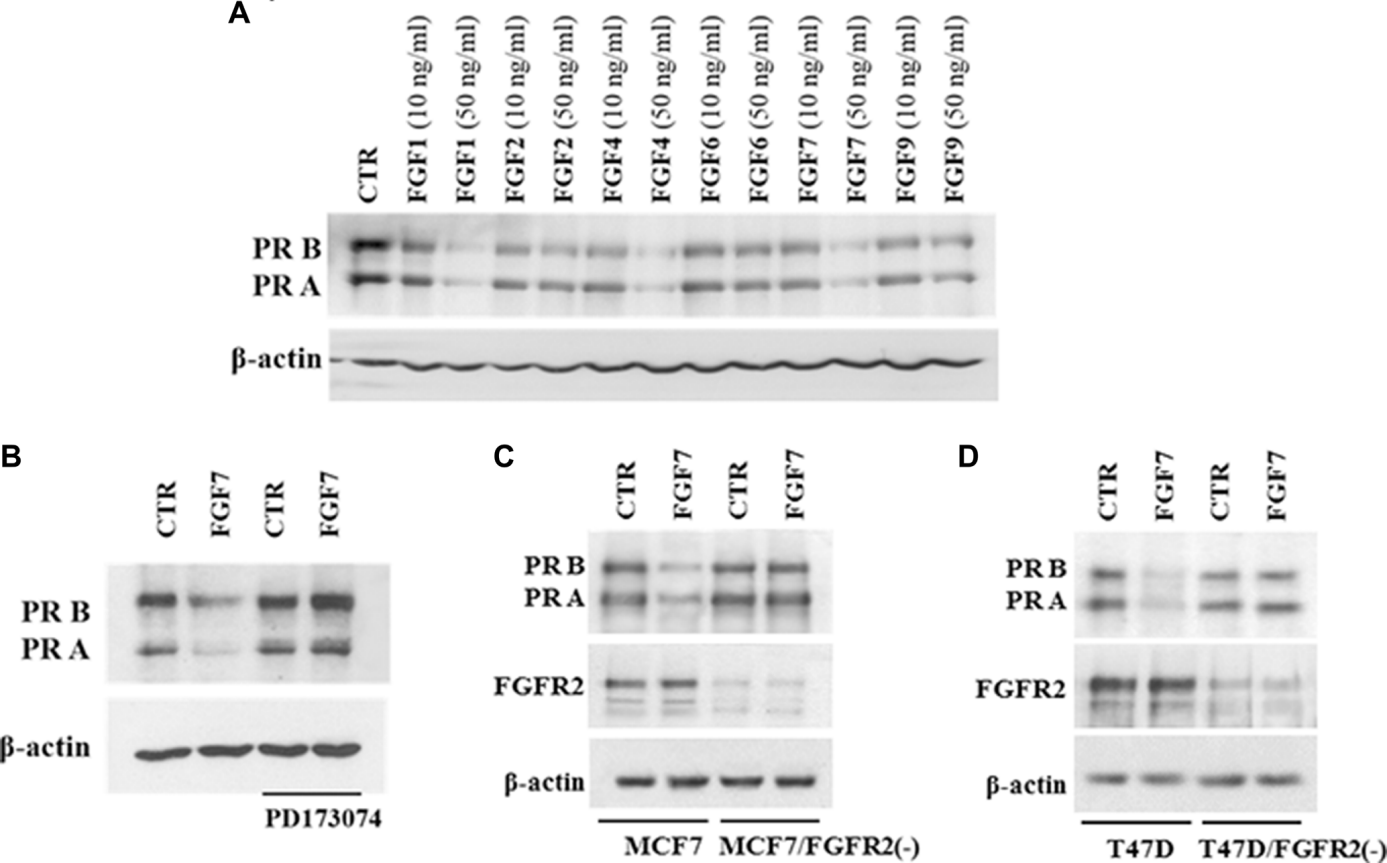
FGF/FGFR signalling downregulates PR (**A**) MCF7 cells were serum starved and treated with a panel of FGFs (10 ng/ml or 50 ng/ml) for 48 hours. PR expression was evaluated by western blotting. (**B**) MCF7 cells were grown with/without FGFR inhibitor (PD173074, 100 nM), stimulated with FGF7 and analysed for PR expression. (**C**–**D**) Knockdown of FGFR2 in MCF7 and T47D cells abolishes FGF7-mediated effects.

A cross-talk between FGFR2 and PR signalling and a nuclear interaction between FGFR-2 and PR in breast cancer cells have already been reported [25]. It has also been demonstrated that activity of various growth factors (e.g. EGF, IGF-1, heregulin) may affect PR protein and/or mRNA levels [24, 26, 31]. Herein we found that prolonged treatment (48 h) of MCF7 BCa cells with various FGFs (FGF1, FGF2, FGF4, FGF6, FGF7 and FGF9) downregulated levels of both PR isoforms (Figure 1A). Since PR A and PR B were equally responsive to the treatment with FGFs (no change in the PR A: PR B ratio was observed), hereafter PR will refer to both isoforms. All tested FGFs affected PR expression. The strongest effect was observed for FGF1, FGF4 and FGF7 (all at 50 ng/ml). Based on this result and published evidence of a role of FGF7 in both physiology and carcinogenesis of the mammary gland [32–34] FGF7 was used for further experiments. An impact of FGF7 on PR expression was confirmed in two other PR-expressing cell lines (T47D and BT474) (Supplementary Figure S1). Similarly to soluble FGFs, cancer-associated fibroblast (CAFs), known to be a rich source of various FGFs (including FGF7 [23]), had an impact on PR expression. MCF7 cells subjected to CAFs-conditioned medium (CAF-CM) displayed a noticeable decrease of PR level (Supplementary Figure S2).

To verify engagement of FGF receptors in FGF7-induced PR downregulation, cells were incubated with PD173074 (a well characterized, specific FGFR inhibitor [35, 36]) and then stimulated with FGF7 (Figure 1B). Pre-treatment with PD173074 nearly completely abolished FGF7-mediated downregulation of PR. Since it is well documented that FGF7 binds with the highest affinity to FGFR2 [37, 38], stable knock-down of *FGFR2* gene was performed to confirm FGFR2 involvement in PR decrease in MCF7 and T47D cells. Results showed that FGFR2 silencing attenuated FGF7-triggered PR loss (Figure 1C–1D). Control experiment with another siRNA (targeting 5′-TTA GTT GAG GAT ACC ACA TTA-3′ in FGFR2 [39]) excluded existence of a possible off-target effect (Supplementary Figure S3). These results indicate that FGF7/FGFR2 activation is involved in regulation of PR level in BCa cells.

### PR is activated in FGF7-initiated signalling

**Figure 2:**
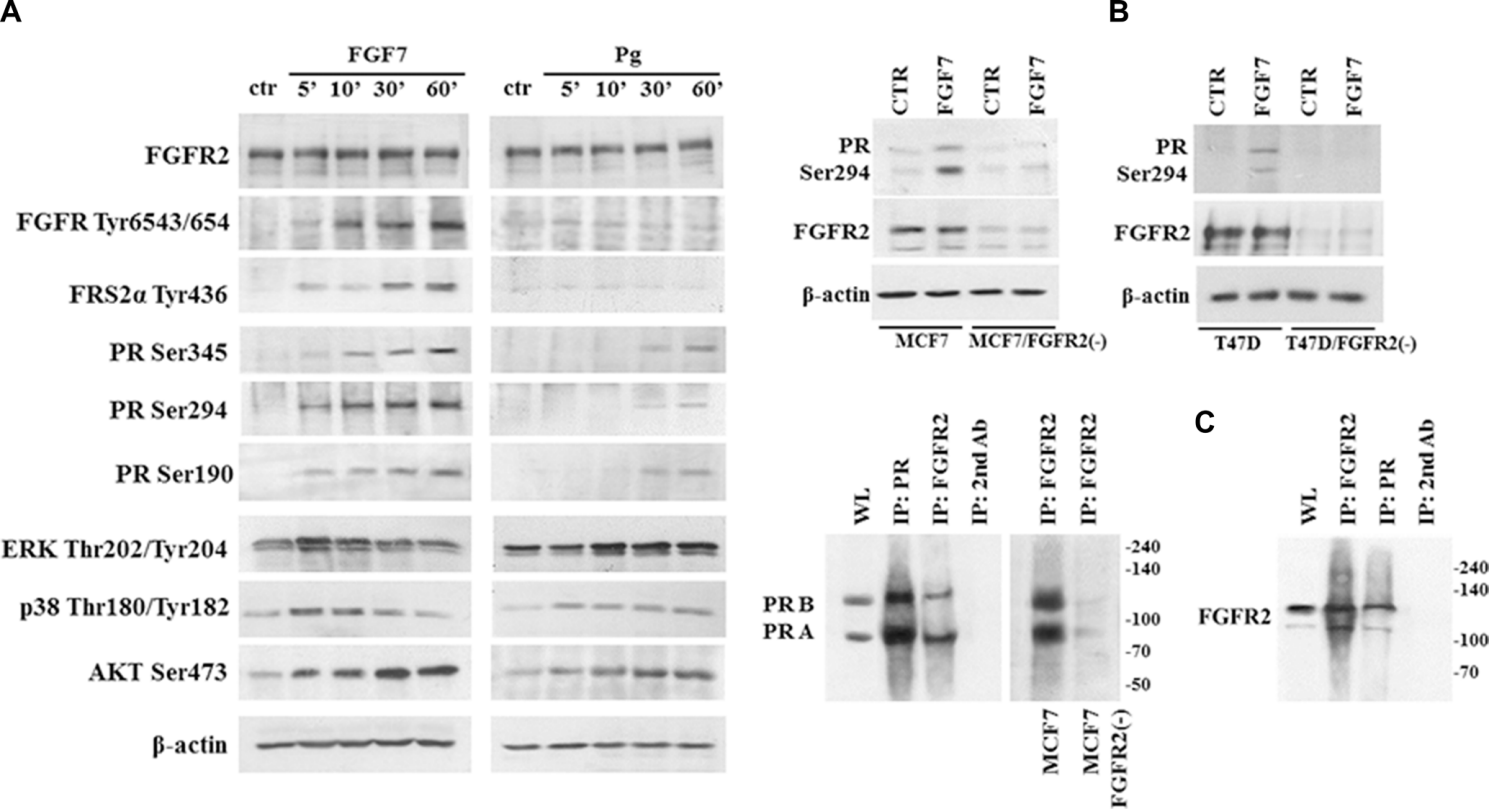
FGF7/FGFR2 activates PR (**A**) PR is phosphorylated upon FGF7 stimulation. MCF7 cells were starved in serum-free media and incubated with FGF7 (50 ng/ml) or Pg (100 nM) for indicated periods of time. (**B**) FGFR2 silencing in MCF7 and T47D cells abolishes FGF7-mediated activation of PR Ser294. (**C**) Interaction between FGFR2 and PR was verified by co-immunoprecipitation. Cell lysis was done in 1% Triton X-100, WL – whole lysate.

Progesterone receptor is activated upon binding of progesterone or its synthetic equivalents. Alternatively, PR activation can be induced independently of Pg through growth factors-related signalling [40]. To determine whether FGF7-triggered cascades affect PR, MCF7 cells were serum-starved and incubated for indicated periods of time with FGF7 or Pg (Figure 2A). As expected, FGF7 induced a gradual increase of phosphorylation of FGFR, Fibroblast Responsive Substrate 2 (FRS2) and AKT. Members of the MAPK family – ERK and p38 reached the peak of activation after 5 min of exposure to FGF7. We also observed that stimulation with FGF7 led to phosphorylation of PR at Ser190, Ser294 and Ser345 as well as rapid (after 5 min) re-localization of cytoplasmic pool of PR to nucleus (Supplementary Figure S4). Interestingly, FGF7-induced phosphorylation of PR and other analysed effectors preceded that triggered by Pg (Figure 2A). FGF7 seems to prime (as shown for other growth factors [41]) PR for Pg action which is reflected in enhanced transcription of *CHN2* and *RGS2* – PR-regulated genes [42] in cells simultaneously treated with FGF7 and Pg (Supplementary Figure S5). Knock-down of FGFR2 in MCF7 and T47D cell lines abolished FGF7-mediated activation of PR at Ser294 site (Figure 2B), confirming FGFR2 involvement in PR activation. Moreover, co-immunoprecipitation in relatively stringent conditions (1% Triton X-100) suggested a possible direct interaction between FGFR2 and PR (Figure 2C), recently reported by Cerliani and co-workers [25].

### FGF7/FGFR2 promotes degradation of PR by 26S proteasome

**Figure 3:**
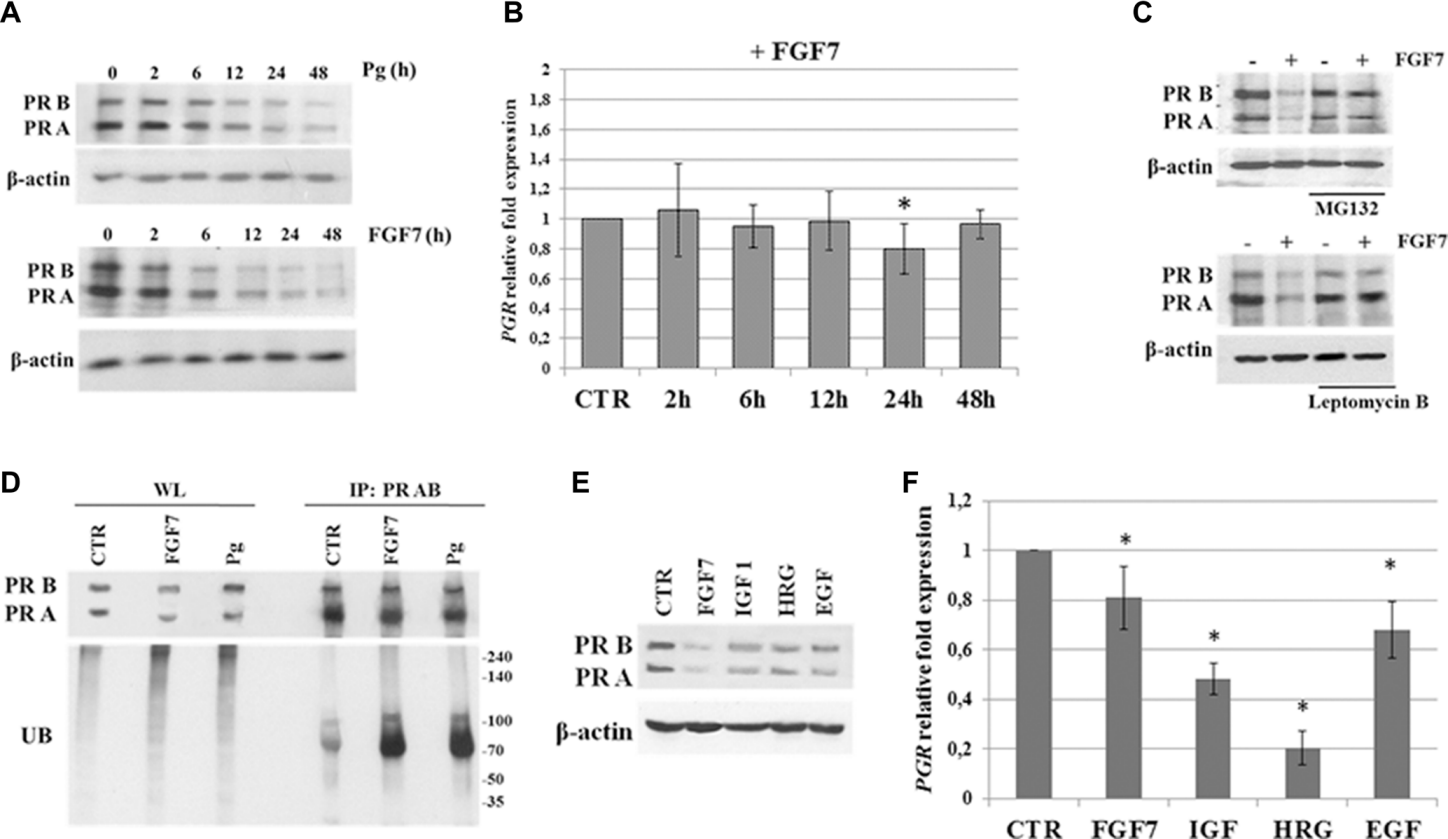
FGF7/FGFR2 promotes PR degradation via proteasome complex (**A**) Various kinetics of PR downregulation upon Pg (100 nM) or FGF7 (50 ng/ml) exposure. Time points as indicated. (**B**) qPCR analysis of *PGR* mRNA level upon treatment with FGF7. (**C**) FGF7 induces proteasomal degradation of PR in cytosol. MCF7 cells were pre-treated with leptomycin B (0.5 nM) or MG132 (2 μM) and incubated with FGF7 (50 ng/ml) for 24 h. (**D**) FGF7 triggers PR ubiquitination. MCF7 cell were serum starved and incubated with FGF7 (50 ng/ml) or Pg (100 nM) for 3 or 4 h, respectively. Cell lysis and immunoprecipitation of PR was done in 1% Triton-X. Ubiquitination level was evaluated by western blot analyses. Protein amount in both lysate (WL) and immunoprecipitated fraction was normalized. (**E**, **F**) Effect of various growth factors on PR downregulation and *PGR* mRNA level (assessed by qPCR), *n* = 3, * *p value* ≤ 0.05.

Previous studies indicated that Pg treatment leads to downregulation of PR via its degradation in the 26S proteasome complex [28, 29, 43, 44]. Interestingly, analysis of PR downregulation kinetics in MCF7 cells revealed decrease of PR protein level faster upon FGF7 than Pg treatment (Figure 3A). A FGF7-triggered, gradually progressing drop in PR level was observed already after 2 h of exposure, while an impact of Pg on PR was first noticed after 12 h of stimulation. On the other hand, FGF7 did not significantly affect *PGR* mRNA expression (Figure 3B). Function of PR is substantially determined by its post-translational modifications (i.e. phosphorylation, acetylation, ubiquitination, sumoylation) [45]. In particular, PR phosphorylation at Ser294 was proved to prime PR for translocation to the nucleus where it acts as a transcription factor. PR then shifts back to the cytoplasm and undergoes ubiquitination which is followed by its degradation in the 26S proteasome complex [43]. Detected discrepancies in kinetics between Pg- and FGF7-induced PR downregulation might, therefore, be due to much faster activation of PR (Figure 2A) (including phosphorylation of Ser294) and subsequent activation of the proteasomal machinery in response to the latter. Kinetics of PR expression levels suggest that downregulation of PR in response to FGF7 does not involve regulation of *PGR* transcription and results only from PR protein degradation.

In order to confirm that the observed FGF7/FGFR2-mediated downregulation of PR (Figure 1C–1D and 3A) was caused by receptor's degradation, MCF7 and T47D cells were incubated with MG132 (a well characterized, specific 26S proteasome inhibitor [46]) or leptomycin B (an inhibitor of nuclear export [47]) and then treated with FGF7 (Figure 3C, Supplementary Figure S6). We noted that an application of either inhibitor completely abolished FGF7-mediated downregulation of PR. In addition, western blot analyses of immunoprecipitated PR revealed that both Pg and FGF7 induced ubiquitination of PR (Figure 3D). Interestingly, the effect of FGF7 was brought about much faster than that induced by Pg (standardization experiment, data not shown), which is in agreement with observed ligand-specific kinetic of PR activation and subsequent degradation (Figure 2A and 3A). These results clearly indicate that FGF7/FGFR2 signalling triggers a cascade of events involving PR phosphorylation, ubiquitination and, eventually, proteasome-dependent receptor's degradation.

It has been reported that PR levels can be affected by other growth factors [24, 48, 49]. Accordingly, cells were incubated for 24 h with IGF-1, heregulin (HRG) and EGF and their effect on PR expression at both protein and mRNA level (Figure 3E–3F), in relation to that induced by FGF7, was assessed by western blotting and qPCR, respectively. All analysed growth factors decreased PR at the protein level but the biggest drop was observed in response to FGF7. Importantly, the level of *PGR* mRNA upon FGF7 and EGF (reported to induce PR proteasomal degradation [29]) treatment was reduced in only 20–30%, whereas IGF and heregulin affected *PGR* mRNA transcription and/or stability resulting in 50–80% reduction in mRNA level. These observations suggest that the effect of FGF7 on PR expression is due to protein degradation rather than downregulation of *PGR* transcription.

### RSK2 mediates FGF7/FGFR2-dependent PR degradation

**Figure 4:**
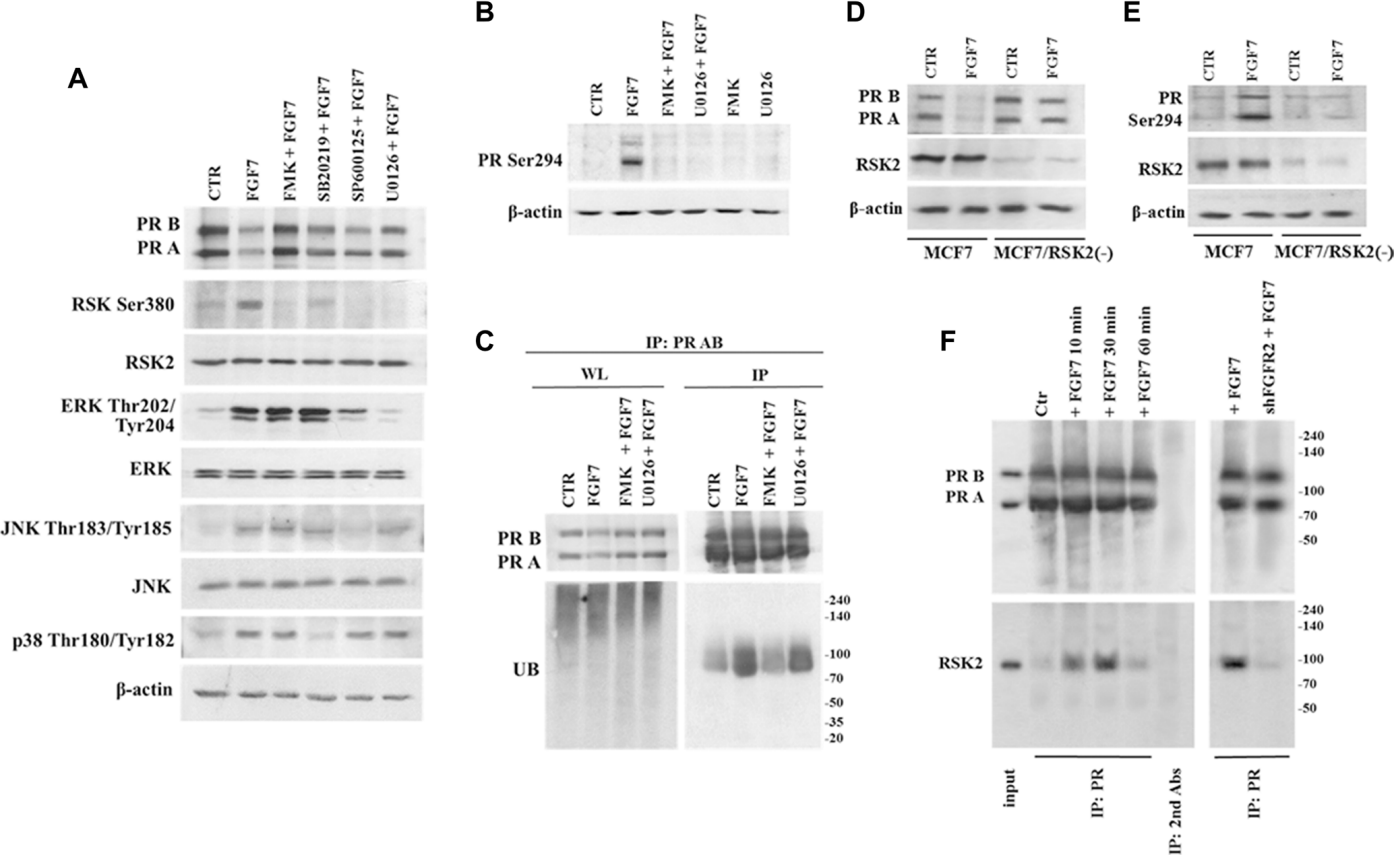
RSK2 mediates FGF7/FGFR2-dependent degradation of PR (**A**) Involvement of various members of MAPK signalling pathway in FGF7/FGFR2-dependent PR degradation. Cells were pre-treated with following inhibitors FMK (10 μM), U0126 (10 μM), SB202190 (10 μM) or SP600125 (10 μM) and stimulated with FGF7 (50 ng/ml) for 24 h. (**B**) RSKs activity is responsible for PR Ser294 phosphorylation (upon 60 min of exposure to FGF7) and (**C**) PR ubiquitination (upon 3 h of exposure to FGF7). Protein amount in both lysate (WL) and immunoprecipitated fraction was normalized. (**D**) Knock-down of RSK2 abrogates PR degradation and (**E**) phosphorylation. (**F**) FGF7/FGR2 signalling results in formation of transient PR/RSK2 complex.

It has been previously shown that MAPK pathway is responsible for PR phosphorylation, an event crucial for receptor's function and stability [18]. In order to identify downstream mediators of MAPK pathway responsible for FGF7/FGFR2-induced degradation of PR we used inhibitors of the following kinases: MEK1/2 (U0126), p38 (SB202190) JNK (SP600125) and, based on our previous study [50], RSKs (FMK). MCF7 cells were pre-incubated with individual compound and then treated with FGF7 (Figure 4A). Application of inhibitors interfered to various degrees with FGF7-dependent effect on PR degradation. Strikingly, inhibition of RSKs completely abolished the observed PR downregulation. Analysis of PR Ser294 phosphorylation (Figure 4B) and receptor's ubiquitination (Figure 4C) revealed that activity of RSKs and, as previously shown ERK [43], is crucial for sequential events leading to PR proteasomal degradation. To verify an involvement of FGFR2-activated RSK2 in PR degradation we silenced RSK2 expression in MCF7 and T47D cell lines. As expected, knock-down of RSK2 affected FGF7-driven PR degradation (Figure 4D, Supplementary Figure S7) as well as PR Ser294 phosphorylation (Figure 4E). On the other hand, overexpression of constitutively active RSK2 [51, 52] did not affect steady-state level of PR but increased FGF7-mediated downregulation of PR. The latter was abolished by inhibitor of either RSK or FGFR (Supplementary Figure S8). These suggest that RSK2 targets PR specifically in the FGFR2 signalling. Co-immunoprecipitation analysis revealed that RSK2 and PR are likely to get into direct, transient interaction upon FGF7/FGFR2 signalling (Figure 4F). Taken together, our findings clearly identify RSK2 as a member of the PR regulatory system.

### FGF7/FGFR2 regulates PR-dependent cell activities

**Figure 5:**
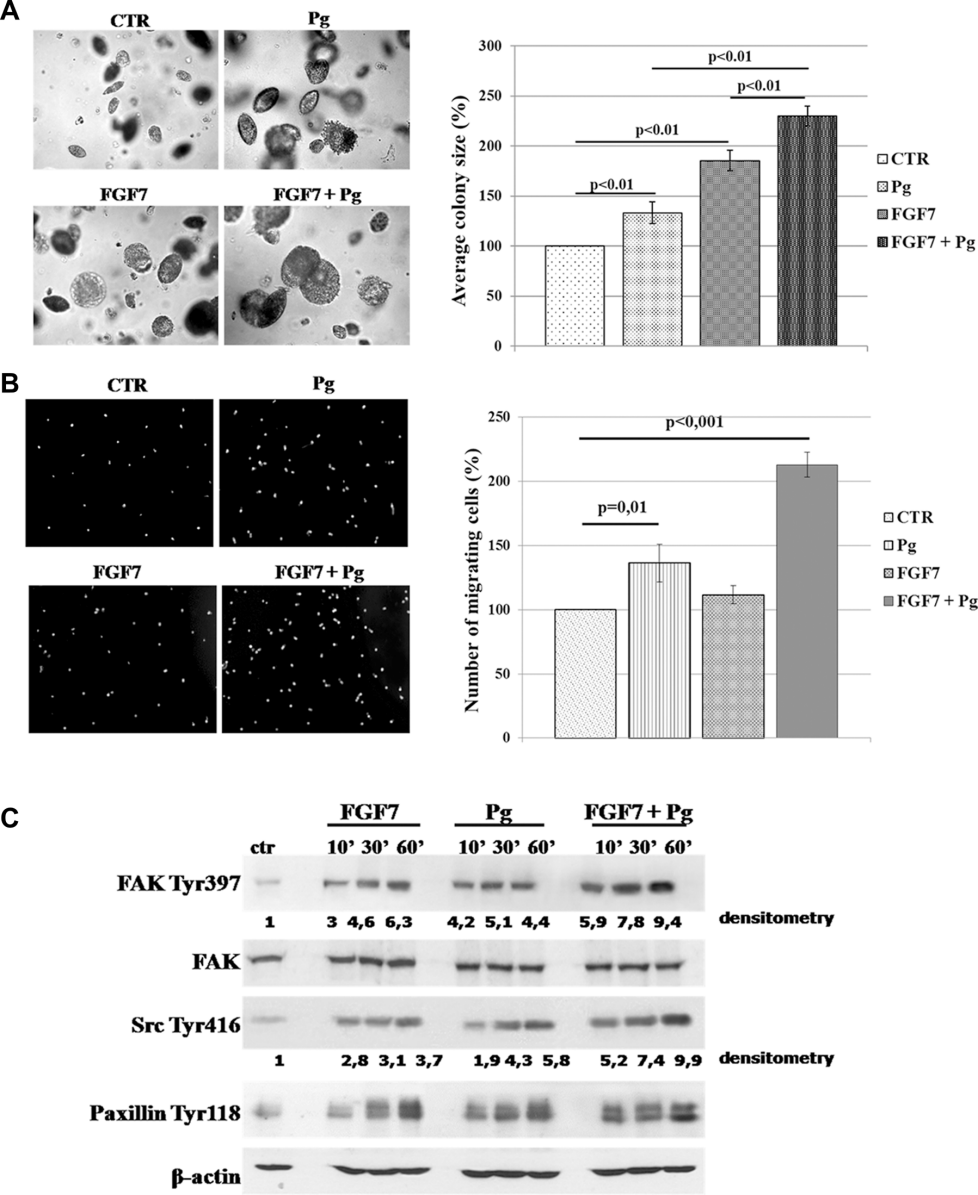
FGF7/FGFR2 regulates PR-mediated cell behaviour (**A**) Synergistic effect of FGF7 and Pg on MCF7 cells growth in soft agarose. MCF7 colonies were cultured in soft agarose in media supplemented with Pg (100 nM) and/or FGF7 (10 ng/ml) for 30 days. The values presented are means ± SD (*n* = 3). (**B**) FGF7 enhances Pg pro-migratory effect. Cells migrated towards medium supplemented with Pg (100 nM) and/or FGF7 (50 ng/ml). The values presented are means ± SD (*n* = 3), *p value* indicated on graphs. (**C**) Activation of FAK, Src and paxillin upon Pg and/or FGF7 treatment. Experiment was highly reproducible. The experiment was done in duplicate.

Previous studies indicated that growth factors exerted a faster and stronger effect on PR-mediated proliferation and migration of BCa cells than that caused by Pg/progestin alone [31, 41]. To investigate functional consequences of FGF7/FGFR2 involvement in PR-related cell behaviour, we evaluated anchorage-independent growth of MCF7 cells upon stimulation with Pg and/or FGF7 (Figure 5A). We found that both Pg and FGF7 promoted cell growth in soft agarose. Importantly, their simultaneous application revealed an additive effect on cell growth. Evaluation of pro-migratory action of FGF7/PR interdependence showed a similar tendency – a cumulative, motility promoting effect of combined FGF7 and Pg (∼2.13–fold vs. ∼1.36-fold for Pg and 1.11 for FGF7 treatment) (Figure 5B). Enhancement of Pg action by FGF7 was additionally confirmed at the molecular level in cells treated with Pg, FGF7 or Pg/FGF7. Both Pg and FGF7 induced phosphorylation of key mediators of cell migration i.e. FAK, Src and paxillin (Figure 5C) but the strongest effect (particularly on Src and FAK, as shown by densitometry - ImageJ software) was observed in cells treated with their combination. These data are in agreement with previously reported sensitization of PR to Pg by growth factors [18, 29, 41].

### Poor prognosis of RSK-P-positive/PR-negative patients

**Figure 6:**
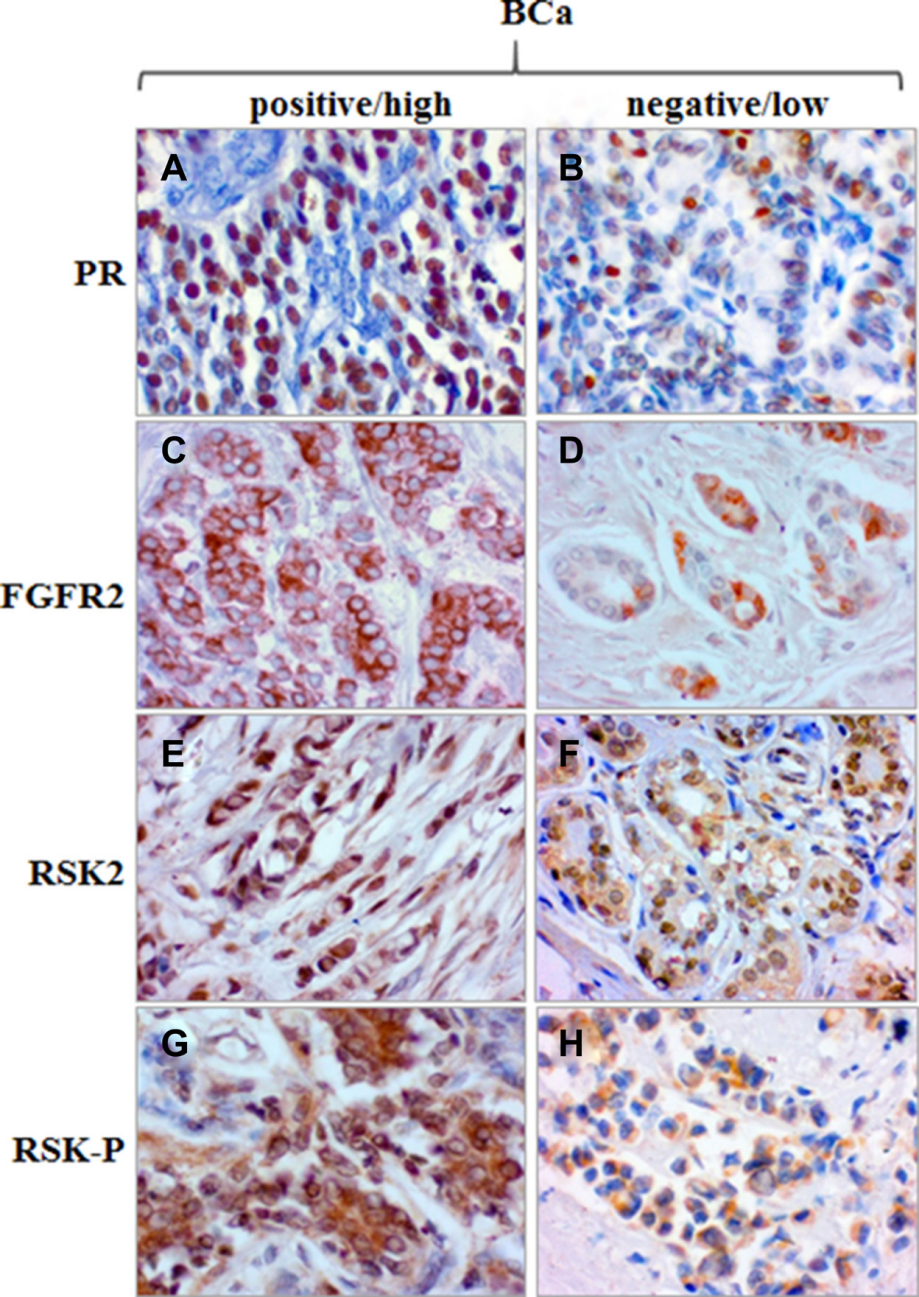
Expression of PR, FGFR2, RSK2 and RSK-P in BCa tissue samples. Examples of high/positive and low/negative immunoreactivity for PR (A/B), FGFR2 (C/D), RSK2 (E/F) and RSK-P (G/H).

**Figure 7:**
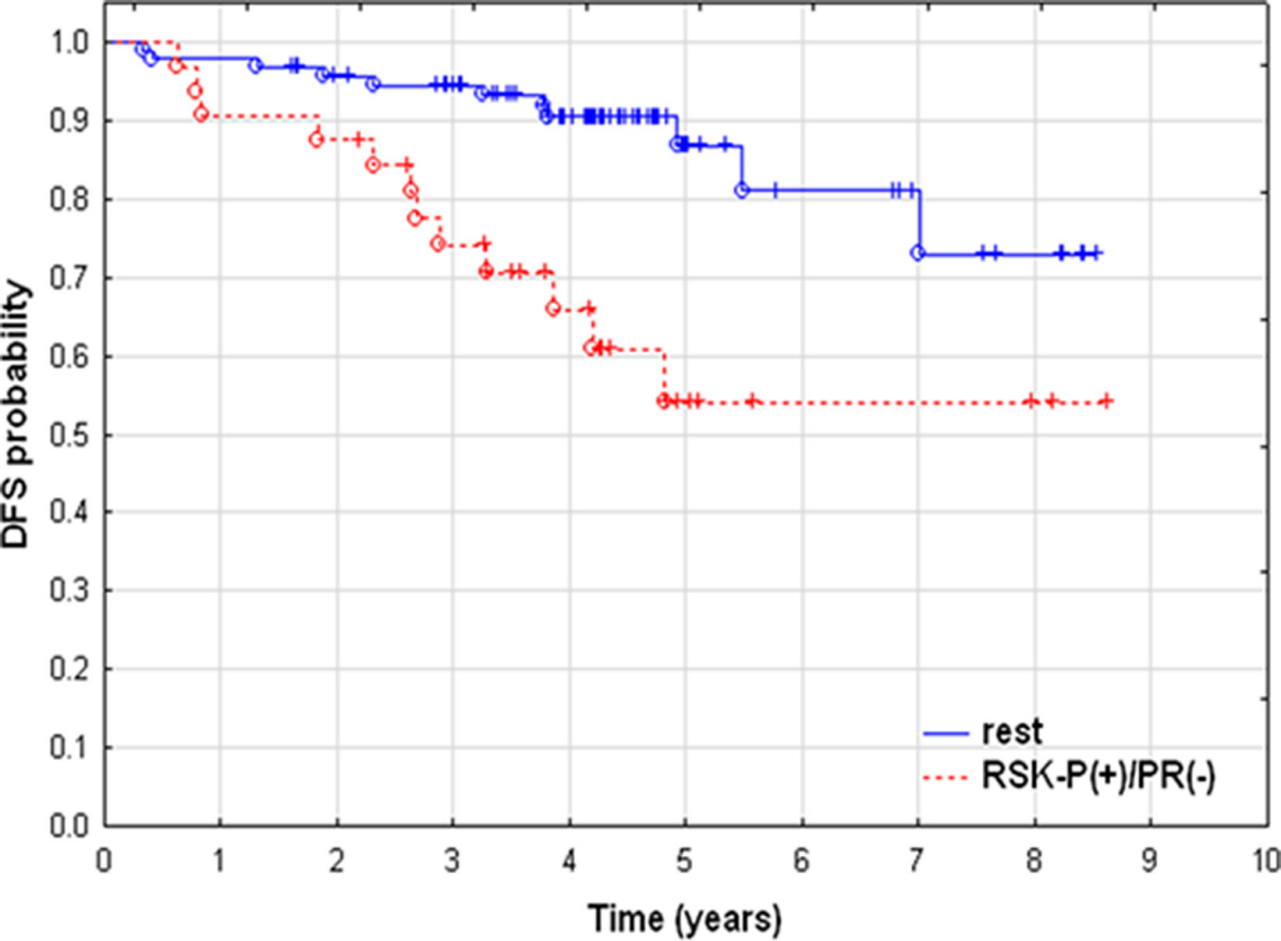
Poor prognosis of RSK-P-positive/PR-negative patients. Kaplan-Meier curves of disease–free survival. Patients with RSK-P(+)/PR(–) (*N* = 32) vs. rest of the cohort (*N* = 93).

We have recently demonstrated that both FGFR2 and RSK2 were expressed in primary breast cancer samples and lack of combined immunoreactivity for FGFR2 and activated RSK (RSK-P) was predictive of a better patients’ disease-free survival [30]. Here, we further evaluated clinical significance of the FGFR2/RSK-P pathway and, in the same group of patients (*N* = 152, Supplementary Table S1), assessed expression of FGFR2, RSK2 and RSK-P in relation to clinicopathological features, and in particular, the PR status. Expression of PR was seen predominantly in the nucleus, whereas the pattern of immunoreactivity for remaining proteins was highly heterogeneous with regards to both cellular localization and level of expression. Examples of levels of high and low expression (classified as positive and negative, respectively) are presented on Figure 6. FGFR2 expression showed, as previously reported [25], a positive, statistically significant association with PR (Table 1, p = 0.000006). There was also a correlation between expression of PR and RSK2 (*p* = 0.023). Importantly, RSK-P showed inverse correlation with PR status (*p* = 0.016), which gives support to the functional significance of identified *in vitro* RSK2-dependent downregulation of PR. Analysis of relationships between the RSK-P(+)/PR(–) phenotype and clinicopathological characteristics revealed statistically significant associations with grade and inverse with ER (Table 2). Patients with RSK-P(+)/PR(–) tumours had 3.629-fold higher risk of recurrence (Table 3, p = 0.002), when compared with the rest of the cohort (Figure 7, p = 0.001). Moreover, RSK-P(+)/PR(–) phenotype was shown an independent marker of recurrence (Table 3, p = 0.006).

**Table 1:** Correlation of PR with FGFR2, RSK2 and RSK-P in BCa

correlation	tau	*p*	*N*
	Kendall		
PR & FGFR2	0.2649	**0.000006**	133
PR & RSK2	0.1377	**0.023**	124
PR & RSK-P	–0.1456	**0.016**	125

Samples analysed by IHC for proteins expression. Numerical values of correlations coefficients (Kendall's tau), *p* values and number of patients are presented in the corresponding boxes.

**Table 2:** Association between RSK-P(+)/PR(–) phenotype and clinicopathological features analysed by IHC in BCa samples

Feature	*P* value
	RSK-P(+)/PR(–)
Tumor size	0.180
Nodal status	0.549
Grade	**0.00005**
HER2	0.075
ER(-)	**0.00012**

Statistically significant *p* values are given in bold.

**Table 3:** Univariate and multivariate analysis of prognostic factors

	UNIVARIATE ANALYSIS DFS			MULTIVARIATE ANALYSIS DFS		
Variable	*N*	Hazard ratio (95% CI)	*p*	*N*	Hazard ratio (95% CI)	*p*
Tumor size (T3–4 vs. T1–2)	151	4.745 (2.220–10.142)	**0.00006**	124	5.830 (2.240–15.170)	0.0003
Nodal status (positive vs. negative)	150	2.373 (1.091–5.162)	**0.029**			NS
ER (positive vs. negative)	149	0.523 (0.257–1.062)	0.073			NS
PR (positive vs. negative)	149	0.367 (0.180–0.750)	**0.006**			–
Grade (G3 vs. G1–2)	132	1.469 (0.652–3.308)	0.353			NS
HER2 status (positive vs. negative)	128	1.348 (0.503–3.612)	0.553			NS
RSK2 (positive vs. negative)	124	0.699 (0.293–1.666)	0.419			NS
RSK-P (positive vs. negative)	127	2.134 (0.790–5.765)	0.135			–
RSK-P(+)/PR(−) vs. rest	125	3.629 (1.599–8.237)	**0.002**	124	3.193 (1.393–7.321)	**0.006**

## DISCUSSION

In response to Pg (or synthetic ligands) binding, PR dimerizes and translocates to the nucleus. It acts there as a transcription factor which is followed by its re-translocation to the cytoplasm and subsequent degradation. Recent data have shown that the PR can also be activated by an alternative mechanism involving growth factor receptors (e.g. EGFR, IGFR, FGFR2)-triggered signalling [24, 25, 53].

It has been well documented that cancer-associated fibroblasts secrete a number of growth factors, including FGFs [23], implicated in cancer progression and lack of responsiveness to treatment [49]. In particular, FGFR2 and its ligands were shown to contribute to BCa development [32, 37, 54], but their possible involvement in the regulation of steroid hormone receptors function still remains poorly understood. Giulianelli and co-workers reported that PR can be activated in response to CAF-secreted FGF2, which may play part in the development of BCa hormone-independence. In a more recent study, they revealed a mechanistic association between FGFR2 and steroid hormone receptor, demonstrating a nuclear interaction between FGFR2 and STAT5, as PR coactivators at the DNA PR-responsive elements [25]. Results of our study showed that stimulation of PR(+) breast cancer cell lines with various FGFs decreased level of PR with the highest impact being observed for FGF1, FGF4, FGF7. As expected, downregulation of PR was also noticed upon treatment with CAF-conditioned medium. There are a number of studies of the role of FGF7 in biology of the mammary gland [32–34]. In light of these findings implicating FGF7 in both physiology and carcinogenesis of the mammary gland, FGF7 has been chosen for our analyses of the interdependence between FGFs/FGFR2 and PR. Binding of FGF7 to the FGFR2, as demonstrated for other FGFs affecting PR level [37], was confirmed in MCF7 cells, where stable knock-down of FGFR2 nearly completely abolished FGF7-mediated downregulation of PR. It is likely, therefore, that the observed effect of CAFs on PR expression was brought about by the FGF7/FGFR2-dependent mechanism.

It has been reported that PR can be activated by growth factors (i.e. EGF, HRG), independently of Pg and, interestingly, much faster than by its cognate ligand [26, 55]. We confirmed these observations and found that in MCF7 cells, FGF7-mediated PR downregulation was indeed more swiftly initiated than the process triggered by Pg. We also verified cross-talk between FGFR and PR signalling and observed phosphorylation of PR in response to treatment with both Pg and FGF7. This is in agreement with previously reported effects of growth factors on PR activation [18, 25, 55]. Surprisingly, we did not observe phosphorylation of FGFR or its direct downstream effector – FRS2α in response to Pg (Figure 2A) demonstrated recently by Cerliani and co-workers [25]. This discrepancy could be due to a different cell line model and/or applied conditions of stimulation (Pg instead of synthetic progestin R5020). However, similarly to Cerliani et al., we confirm a possible direct interaction between FGFR2 and PR.

Phosphorylation of PR at Ser294 is considered to prime PR for translocation to the nucleus, increase PR transcriptional activity and trigger PR ubiquitination with subsequent degradation in the cytoplasm [18]. Interestingly, we observed FGF7/FGFR2-dependent activation of PR at Ser294. This suggests that downregulation of PR triggered by FGF7 is associated with receptor degradation rather than regulation of *PGR* transcription. This has been verified by application of MG132 - 26S proteasome inhibitor and Leptomycin B - nuclear export inhibitor, which both nearly completely abrogated FGF7-mediated PR downregulation. Analyses of an impact of FGF7 *vs*. other growth factors (e.g. IGF-1, HRG or EGF) on PR protein and *PGR* mRNA level showed that FGF7 affected mainly the former with the weakest effect, of all tested GFs, on *PGR* mRNA expression. We also found that stimulation with FGF7 enhanced the process of PR ubiquitination to the same extent as did the Pg treatment. Interestingly, we observed that ubiquitination of PR proceeded faster in response to FGF7 than Pg, which may be a result of presented differences in kinetics of PR activation triggered by these two factors.

Molecular mediators of the FGF7/FGFR2-initiated PR degradation have not been unequivocally recognised yet. However, MAPK pathway was strongly suggested to regulate PR activity, including Ser294 phosphorylation known to be indispensable for PR degradation [43]. Our previous studies identified RSK2 as a downstream target of FGF2/FGFR2 signalling pathway [50]. Importantly, RSK2 was demonstrated to closely relate to MSK1/2, proved to interact with and activate PR [56]. Application of various inhibitors of individual members of the MAPK family indicated, as previously reported [57], that p38 and ERK activity was involved in PR degradation. Importantly, in our analyses, the strongest inhibitory effect, nearly completely abolishing FGF7-mediated PR degradation, was noted in cells pre-incubated with FMK (RSKs inhibitor). Inhibition of RSKs elevated PR level, even in control (non-treated) cells, which may suggest a primary role of RSKs in regulation of PR function. In addition, RSKs seemed to be involved in PR phosphorylation at Ser294 and receptor's ubiquitination in response to FGF7. We also identified RSK2, as a member of the RSK family important for regulation of PR function. Co-immunoprecipitation suggested a possible direct interaction between RSK2 and PR upon FGF7/FGFR2 signalling. As, RSK2 was previously shown to activate and interact with estrogen receptor [58], it may indicate RSK2 as an essential mediator of a likely universal mechanism of regulation of a steroid hormone receptors activity.

Evaluation of cellular effects of the cross-talk between FGF7/FGFR2 and PR revealed that FGF7 or Pg, tested separately, stimulated anchorage-independent growth and migration of breast cancer cells. Combination of both agents disclosed a clear synergism between them, reflected by enhancement of cell growth and motility. Analysis of activation of proteins directly involved in regulation of cell migration (i.e. FAK, Src, paxillin) upon cells’ treatment with FGF7 and/or Pg suggested they may have exerted a cumulative biological effect. As previously demonstrated [18], these data together with analyses of expression of PR-dependent genes upon FGF and/or Pg stimulation (Supplementary Figure S5) strongly imply that PR, primed by growth factors (including FGF7), may respond more efficiently to its steroid cognate ligand.

Clinical significance of FGFR2-RSK2 signalling pathway was analysed in breast cancer samples in relation to the PR status. Results demonstrated that positivity for RSK-P identified a subgroup of patients with PR-negative BCa with increased risk of recurrence. Furthermore, RSK-P(+)/PR(–) phenotype was found an independent marker of poor disease-free survival. In addition, PR expression inversely correlated with RSK-P, which is in agreement with our *in vitro* observations and gives weight to biological meaning of investigated molecular mechanisms. Taken together, our study demonstrated for the first time that the FGF7/FGFR2-triggered signalling pathway, involving RSK2 activity and targeting PR, may be a new mechanism of breast cancer progression in response to stromal (e.g. cancer-associated fibroblasts) stimuli toward steroid hormone negative BCa.

## MATERIALS AND METHODS

### Cell lines, antibodies, reagents

MCF7, T47D and BT474 cell lines were obtained from ATCC. Cell lines were passaged for a maximum of 3–4 months post resuscitation and regularly tested for mycoplasma contamination. MCF7 and T47D cells were routinely maintained in DMEM, BT474 in RPMI supplemented with insulin (5 μg/ml). All media contained 10% FBS and penicillin/streptomycin (100 U/ml/ 100 μg/ml). For analyses of PR function, phenol red-free media and dextran charcoal-treated FBS were used. All media and supplements were purchased from Sigma-Aldrich or HyClone. The following antibodies were obtained from Santa Cruz Biotechnology: anti-FAK (sc-558), anti-FGFR2 (sc-122), anti-PR (sc-7208), anti-Ub (sc-8017). Antibody against β-actin (A5316) was obtained from Sigma-Aldrich. All the remaining antibodies were from Cell Signaling Technology: anti-AKT (#9272), anti-AKT-Ser473 (#4048), anti-ERK1/2-Thr202/Tyr204 (#9101), anti-FAK-Tyr397 (#3283), anti-FGFR-Tyr653/654 (#3471), anti-FRS2α-Tyr436 (#3861), JNK-Thr183/Tyr185 (#9251), anti-p38-Thr180/Tyr182 (#9211), anti-paxillin-Tyr118 (#2541), anti-PR-Ser190 (#3171), anti-PR-Ser345 (#12783), anti-RSK2 (#5528), anti-RSK-Ser380 (#9335), anti-RSK-Thr359/Ser363 (#9344), anti-Src-Tyr416 (#6943). Anti-PR-Ser294 antibodies were kindly provided by Dr Carol Lange (University of Minnesota). All growth factors were purchased from PeproTech. Heparin sodium salt and inhibitors: PD173074, U0126, SB20219, SP600125, FMK, MG132, Leptomycin B were from Sigma-Aldrich.

### Western blotting and immunoprecipitation

Cells were harvested at 60–70% of confluency in cold PBS and lysed with Laemmli buffer (2x concentrated) supplement by 2 mM PMSF, 10 μg/ml aprotinin, 10 μg/ml leupeptin, 5 mM EGTA, 1 mM EDTA, 2 mM Na_4_P_2_O_7_, 5 mM NaF and 5 mM Na_3_VO_4_. Samples containing equal amounts of protein per lane were loaded, resolved in SDS–PAGE and then transferred onto nitrocellulose membrane. The membranes were incubated for 1 h in 5% skimmed milk and probed overnight with specific primary antibodies at 4°C. Secondary antibodies conjugated with HRP (Sigma-Aldrich) and Western Lightning Plus-ECL (PerkinElmer) were used to visualize specific proteins. For immunoprecipitation experiments, cells were lysed in lysis buffer containing 1% Triton X-100. Supernatant was incubated with 2 μg of appropriate antibody overnight at 4°C. The samples were incubated with protein A or protein G beads (Santa Cruz Biotechnology, sc-2001, sc-2002) according to the manufacturer's instructions. Immunocomplexes were eluted from beads with Laemmli buffer and analysed by western blotting.

### qPCR

RNA was isolated with TriPURE reagent (Roche) according to the manufacturer's protocol. Reverse transcription with random hexamer primers was done with Transcriptor cDNA First Strand Synthesis Kit (Roche). *PGR* gene and PR-dependent genes expression analyses were carried out with TaqMan (Applied Biosystem) probes -*PGR* (Hs01556702_m1), *CHN2* (Hs00906968_m1), *RGS2* (Hs01009070_g1) with *ACTB* (Hs99999903_m1) and *GAPDH* (Hs99999005_m1) used as reference genes. Twenty microliter reactions were conducted applying TaqMan Universal PCR Master Mix (Applied Biosystem) on 96-well plates in CFX96 cycler (Bio-Rad, Hercules). Reactions were done in duplicates. Each plate contained an inter-run calibrator - a set of non-template controls and controls for cDNA contamination. Gene expression was calculated using a modified ΔΔC approach, as previously described [59].

### Soft agarose assay for anchorage-independent growth

The 5 × 10^4^ cells per well of 6-well plate were re-suspended in 3 ml of 0.4% low gelling temperature agarose (Sigma) prepared in DMEM containing 10% FBS and overlaid on 3 ml solidified 0.5% agarose made up in the same medium. The top layer was covered with 3 ml of regular medium supplemented, when appropriate, with FGF7 (10 ng/ml) and/or Pg (100 nM). The medium was refreshed every 3 days. After 21 days of culture, colonies were counted and measured with ZEISS PrimoVert microscope and ImageJ software.

### Migration assay

Cell migration was assessed as previously described [60]. Briefly, MCF7 cells were serum-starved overnight. Next day cells were detached with enzyme-free cell dissociation buffer (Millipore) and 1.5 × 10^5^ cells resuspended in serum-free DMEM. The polycarbonate membranes (8 μm pores, BD Bioscience) of inserts were coated with high concentration Matrigel (BD Bioscience) diluted in serum-free DMEM (1:1000). Cells were placed in the inner compartment of Boyden chamber inserts and allowed to migrate for 24 h toward DMEM (10% FBS) ± 50 ng/ml FGF7 and/or 100 nM Pg. Non-migrated cells were removed by cotton swab. Membranes were mounted onto glass slides, cells stained with DAPI and counted in 20 random fields (100x) under AxioVert 200 fluorescent microscope.

### FGFR2 knock-down, RSK2 knock-down, overexpression of RSK2

MCF7/FGFR2(-) and MCF7/RSK2(-) cell lines were generated with lentiviral system based on pLKO.1-TRC vector (Addgene, #10878) with cloned shRNA designed on the basis of the following siRNA sequences: FGFR2 5′-GAG AUU UGG UAU UUG GUU GGU GGC –3′ [61], RSK2 5′-UUG CUG UCC AUU CUC AGC GCU–3′ [62]. Overexpression of RSK2 was generated with pWZL Neo Myr Flag RPSK6A3 plasmid (Addgene, #20627). Transfection was done with TurboFect Transfection Reagent (Thermo Scientific) according to the manufacturer's protocol. Stable cell line expressing constitutively active RSK2 were established by neomycin (Sigma Aldrich) selection.

### Stimulation with growth factors, inhibitors effect, signalling analyses

For analysis of growth factors-triggered signalling, cells were serum starved overnight before growth factors were added. Cells were stimulated with various FGFs at 10 or 50 ng/ml and/or Pg (100 nM) for indicated periods of time. In all experiments, FGFs were used together with heparin sulphate (10 ng/ml). When required, media were supplemented with an appropriate inhibitor: PD173074 (100 nM), MG132 (2 μM), leptomycin B (0.5 nM), FMK (10 μM), U0126 (10 μM), SB202190 (10 μM) or SP600125 (10 μM).

### Clinical data

BCa primary tumour samples were obtained from 152 patients treated between 1999 and 2009 at the Medical University of Gdansk Hospital. Follow-up data were available in 147 cases. Clinicopathological characteristics of the cohort are summarized in Supplementary Table S1. ER/PR/HER2 status was determined by routine histopathological assessment. Serial 5 μm paraffin sections of formalin-fixed blocks were processed for immunohistochemistry for FGFR2 (mouse anti-human; 1:600; Abnova #H00002263-M01), RSK2 (rabbit anti-human; 1:200; Life Span BioSciences, # LS-B7708) and phospho-RSK (RSK-P) (rabbit anti-human; 1:100, Sigma-Aldrich #SAB4503961) using protocols recommended by the manufacturers. As a negative control for immunostaining, primary antibodies were replaced by non-immune sera. Scoring of immunostaining (not distinction was made between subcellular distributions) was carried out as follows: i) 0/negative – no reactivity or only faint reactivity in < 10% of tumour cells; ii) 1+/negative – faint reactivity in ≥ 10% of tumour cells; iii) 2+/positive – weak to moderate reactivity in ≥ 10% of tumour cells; iv) 3+/positive – strong reactivity in ≥ 10% of the tumour cells. Immunohistochemical staining was evaluated and scored independently by two observers (HMR and RK). The agreement on staining intensity was > 90%. Where there was disagreement, intensity was determined by consensus. Final scores were dichotomized into: a) ‘negative/low’ for 0–1 and b) ‘positive/high’ for 2–3. The study was conducted in accordance with the Declaration of Helsinki and approved by the Local Research Ethics Committee of the Medical University of Gdansk (project licence #118/2014/NKBBN).

### Statistical analyses

All statistical analyses were performed using the STATISTICA software (version 10, StatSoft). Kendall's tau rank correlation test was used to study correlation between levels of PR, FGFR2, RSK2 and RSK-P in cancer tissue. Disease-free survival (DFS) was computed using Kaplan-Meier method and compared using log-rank test. Cox proportional hazards regression model was used to identify the independent predictors of DFS. Two-sided *p value* < 0.05 was considered as significant. All *in vitro* experiments were performed in triplicate unless otherwise indicated.

## SUPPLEMENTARY MATERIALS FIGURES AND TABLES


