# Supplementary material for: Fibroblast growth factor signalling induces loss of progesterone receptor in breast cancer cells
Source: Oncotarget. 2016 Nov 12;7(52):86011–25. doi: 10.18632/oncotarget.13322 (PMC5349893; doi:10.18632/oncotarget.13322)
Supplement: Supplementary file 1 [file oncotarget-07-86011-s001.pdf]

Fibroblast growth factor signalling induces loss of progesterone receptor in breast cancer cells

Supplementary Materials

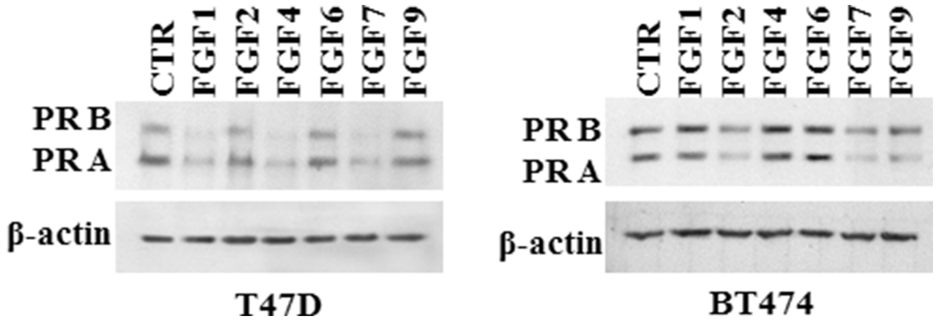

Supplementary Figure S1: FGF/FGFR signalling downregulates PR in BT474 and T47D cells. BT474 and T47D cells were serum starved and treated with FGF1, FGF2, FGF4, FGF6, FGF7 and FGF9 (50 ng/ml) for 48 hours. PR expression was evaluated by western blotting.

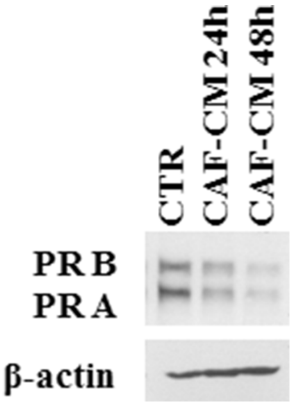

Supplementary Figure S2: Cancer-associated fibroblasts-conditioned medium (CAF-CM) triggers PR downregulation in MCF7 cells.

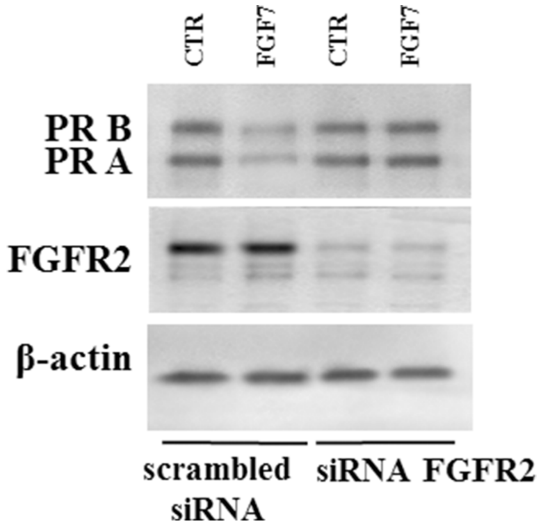

Supplementary Figure S3: FGFR2 mediates FGF7 signalling towards downregulation of PR. Cells were transfected with siRNA targeting 5′-TTA GTT GAG GAT ACC ACA TTA-3′ in *FGFR2* [39] or scrambled siRNA, treated with FGF7 and analysed for PR expression. The experiment was done in duplicate.

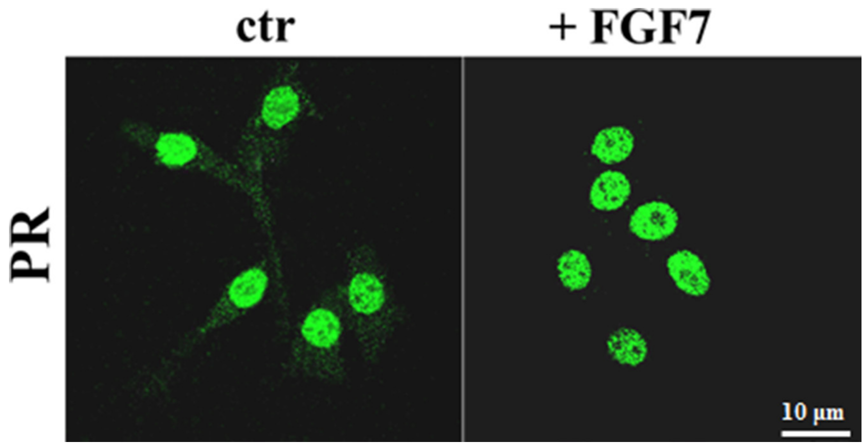

Supplementary Figure S4: FGF7 triggers rapid re-localization of cytoplasmic pool of PR to nucleus. Cells were serum starved and stimulated with FGF7 for 5 min. Representative pictures were taken under confocal microscope.

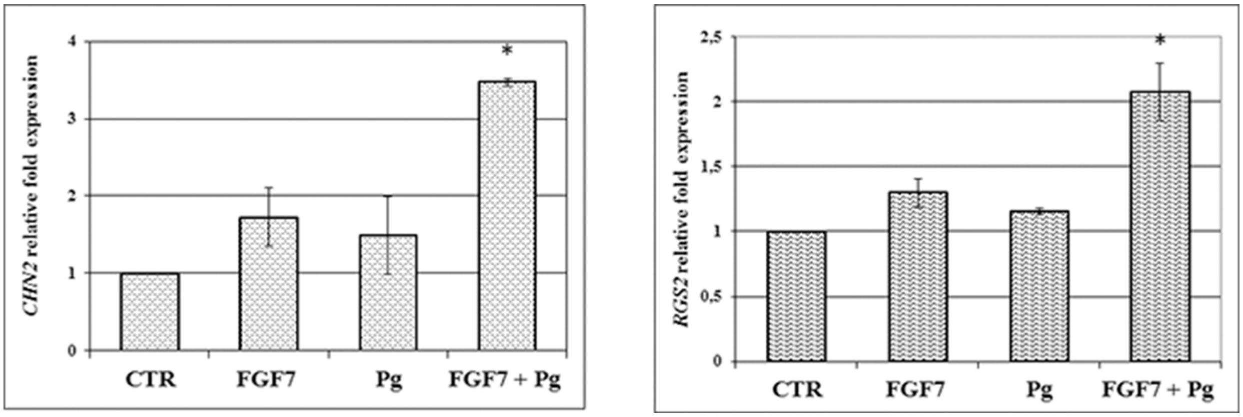

Supplementary Figure S5: qPCR analysis of *CHN2* and *RGS2* - PR-dependent genes expression upon FGF7 and/or Pg treatment (24 h), $n = 3$, $*p \leq 0.01$.

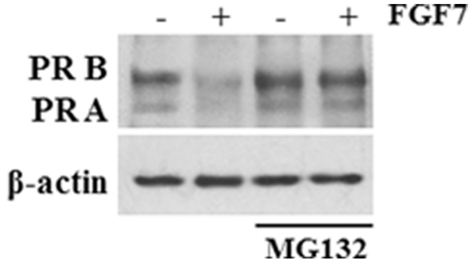

Supplementary Figure S6: Inhibition of proteasomal complex by MG132 abrogates FGF7-driven PR loss in T47D cells. The experiment was done in duplicate.

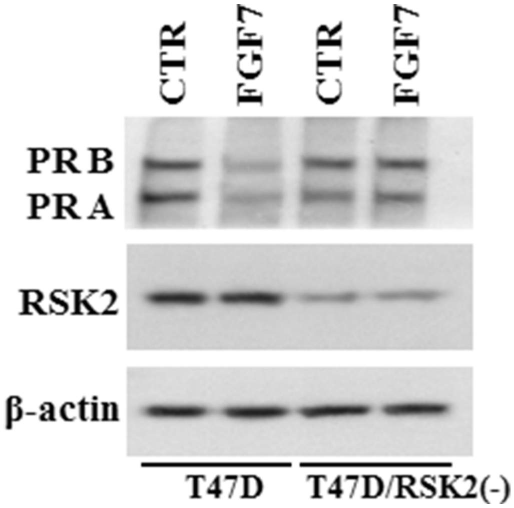

Supplementary Figure S7: RSK2 knock-down abolishes FGF7-driven PR loss in T47D cells. The experiment was done in duplicate.

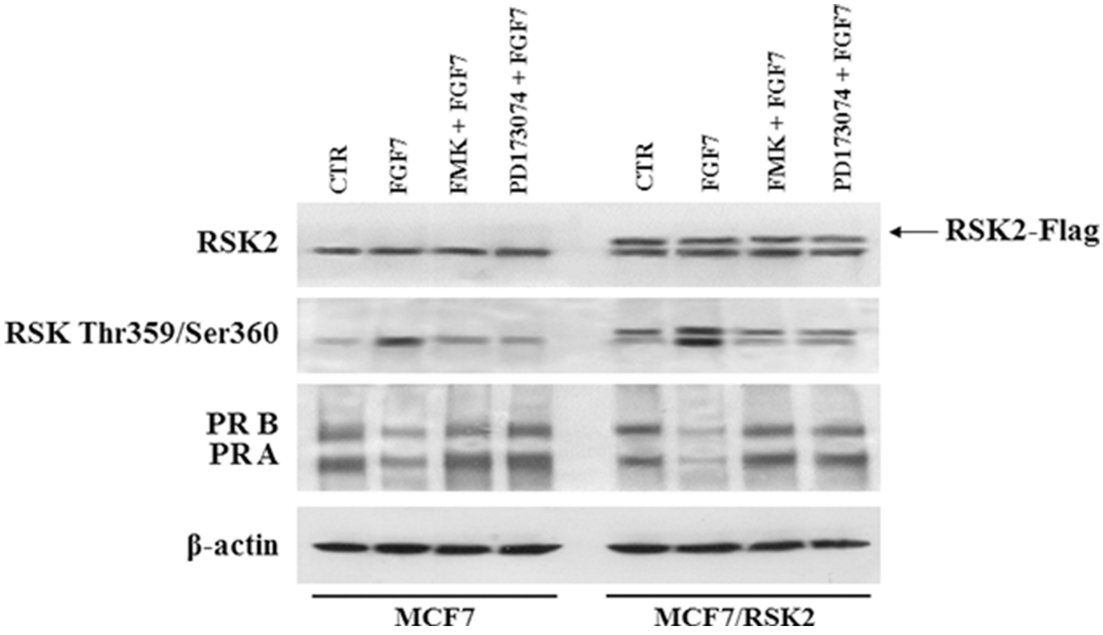

Supplementary Figure S8: RSK2 mediates PR downregulation specifically in FGF7/FGFR2 signalling. Constitutively active RSK2 (Flag-tagged) was expressed in MCF7 cells.

Supplementary Table S1: Patient`s characteristics

Patients' characteristics (N = 152)		
**Age**	**27–86 (average: 58)**	
	N	**%**
**T stage**		
T1	53	34.87%
T2	78	51.32%
T3	9	5.92%
T4	11	7.24%
Missing data	1	0.66%
**N stage**		
N0	68	44.74%
N1	50	32.89%
N2	27	17.76%
N3	5	3.29%
Missing data	2	1.32%
**Grade**		
G1	9	5.92%
G2	80	52.63%
G3	45	29.61%
Missing data	18	11.84%
**HER2**		
Negative	107	70.39%
Positive	21	13.82%
Missing data	24	15.79%
**ER**		0.00%
Negative	63	41.45%
Positive	86	56.58%
Missing data	3	1.97%
**PR**		
Negative	54	35.53%
Positive	95	62.50%
Missing data	3	1.97%
**Histological type**		
Ductal	104	68.42%
Lobular	21	13.82%
Other	14	9.21%
Missing data	13	8.55%
**Molecular subtype**		
HR+, HER2–	83	54.61%
HR+, HER2+	9	5.92%
HR–, HER2+	14	9.21%
TNBC	21	13.82%
Missing data	25	16.45%
